# Assessing the Impact of Case Sensitivity and Term Information Gain on Biomedical Concept Recognition

**DOI:** 10.1371/journal.pone.0119091

**Published:** 2015-03-19

**Authors:** Tudor Groza, Karin Verspoor

**Affiliations:** 1 School of Information Technology and Electrical Engineering, The University of Queensland, St Lucia, Australia; 2 Department of Computing and Information Systems, The University of Melbourne, Melbourne, Australia; 3 Health and Biomedical Informatics Centre, The University of Melbourne, Melbourne, Australia; University of Vermont, UNITED STATES

## Abstract

Concept recognition (CR) is a foundational task in the biomedical domain. It supports the important process of transforming unstructured resources into structured knowledge. To date, several CR approaches have been proposed, most of which focus on a particular set of biomedical ontologies. Their underlying mechanisms vary from shallow natural language processing and dictionary lookup to specialized machine learning modules. However, no prior approach considers the case sensitivity characteristics and the term distribution of the underlying ontology on the CR process. This article proposes a framework that models the CR process as an information retrieval task in which both case sensitivity and the information gain associated with tokens in lexical representations (e.g., term labels, synonyms) are central components of a strategy for generating term variants. The case sensitivity of a given ontology is assessed based on the distribution of so-called case sensitive tokens in its terms, while information gain is modelled using a combination of divergence from randomness and mutual information. An extensive evaluation has been carried out using the CRAFT corpus. Experimental results show that case sensitivity awareness leads to an increase of up to 0.07 F1 against a non-case sensitive baseline on the Protein Ontology and GO Cellular Component. Similarly, the use of information gain leads to an increase of up to 0.06 F1 against a standard baseline in the case of GO Biological Process and Molecular Function and GO Cellular Component. Overall, subject to the underlying token distribution, these methods lead to valid complementary strategies for augmenting term label sets to improve concept recognition.

## Introduction

The latest advances in high-throughput methods in the biomedical field have led to an explosion of publicly available data, much of which has been published in free text form, i.e., manuscripts, technical reports, etc. This vast amount of data makes manual curation of biological entities (e.g., genes, proteins) infeasible [1]. Hence, effort has shifted towards developing automated solutions to support experts in the curation task. In particular, named entity recognition (NER) aims to detect mentions of entities of interest within unstructured textual sources. A large number of approaches have been proposed [2] and the current state of the art places NER at the foundation of many free text processing tasks. The technologies underpinning existing solutions range from rules and dictionaries [3] to machine learning [4], and lately, combinations of these into ensembles or hybrid approaches [5].

In parallel to text mining, in the past decade, ontologies have become central to defining, representing and storing biomedical concepts. As a natural evolution, the traditional NER task has expanded to target detection of ontological concepts, where text spans are associated to ontological entities. Research in this area is referred to as concept extraction or *concept recognition* (CR), and is summarised in [6].

The main challenges associated with CR, most also encountered in NER, are: (i) spelling diversity: “*N-acetylcysteine*” also spelled as “*N-acetyl-cysteine*” or “*NAcetylCysteine*”; (ii) ambiguity: “*star*” may denote a protein (i.e., “*steroidogenic acute regulatory protein*” in mouse), a bone dysplasia syndrome or even the celestial body; similarly, “*long bones*” may refer to an anatomical entity, as well as to an abnormality; (iii) descriptiveness: “*curved femora with rounded distal epiphyses*”; (iv) lack of synonyms: missing term variants—See [7] for a comprehensive analysis of the impact of undocumented synonyms on concept recognition; and (v) terminology gaps: terms that should have been defined by a target ontology and yet are absent.

The literature consists of multiple CR approaches, some relying on direct dictionary lookup combined with stemming and word permutation algorithms—e.g., the NCBO Annotator [8]—others using standard shallow natural language processing (NLP) pipelines (i.e., sentence splitting, tokenization, POS tagging, etc.) augmented with specialised CR modules (applying direct dictionary lookup or machine learning based on dictionaries)—e.g., Neji [9]. Finally, from an ontological perspective, CR systems can be split into two groups: (i) ontology-agnostic—i.e., systems designed to perform CR using any given ontology (e.g., NCBO Annotator or Neji), and (ii) ontology-specific—i.e., systems tailored to perform CR using a specific ontology or set of ontologies—e.g., cTakes [10] or MetaMap [11].

The recent release of the CRAFT corpus [12] has enabled the community to evaluate these approaches in a standardised manner. The CRAFT corpus consists of 67 full-text articles manually annotated with six biomedical ontologies and terminological resources, including Cell Ontology, Gene Ontology or Sequence Ontology. Consequently, we can now observe that the state of the art evaluation results, as reported by [9] and [13], vary significantly with the underlying ontology used for testing and with the matching strategy. Current systems perform extremely well on ontologies comprising simpler concepts. For instance, Neji [9] reaches 0.87 F1 score on the NCBI Taxonomy (exact matching), and the ConceptMapper [14] achieves 0.83 F1 score on the Cell Ontology (exact matching). Their performance decreases substantially on more complex concepts, such as GO biological processes and molecular functions, where Neji achieves 0.35 F1 score, and chemical entities (ChEBI), where ConceptMapper and the NCBO Annotator [8] achieve 0.56 F1 score.

We propose that CR performance can be improved if: (i) case sensitivity is taken into account, and (ii) the ontological concepts are pre-processed by understanding the information gain brought by diverse terms present in their labels.

The absence of case sensitive processing is likely to generate false positives in the presence of ambiguity. For example, the noun “*step*” can be found in 31 of the 67 CRAFT publications and is very likely to be associated with the term PR:000013460 (“*STEP*”, i.e., “*striatum-enriched protein-tyrosine phosphatase*”) if the CR process is performed in a case insensitive manner.

Our second proposal is related to the possible disconnect between ontological concept labels and natural expression of those concepts. For example, Gene Ontology defines GO:0000009 as “*alpha-1, 6-mannosyltransferase activity*”. However, this concept is less likely to be found in text with its complete label and more likely to be encountered only as “*alpha-1, 6-mannosyltransferase*”. As such, from a concept recognition perspective, the token “*activity*” does not bring any added value to this label (since it is present in more than 25,000 other labels or synonyms in GO), but it does impact negatively on the exact matching strategy of CR. Similarly uninformative tokens can be encountered in other ontologies—e.g., “*protein*” and “*complex*” in the Protein Ontology. Prior work noticed this issue, and suggested that a weighting scheme taking into consideration the terminological structure of the underlying ontology might help [15]. We develop such a scheme.

In this paper, we propose an approach to CR that automatically detects the need for case sensitive processing of a given ontology, and quantifies the importance of individual tokens composing ontology concept labels, in order to generate alternate labels for ontology concepts and thereby improve concept recognition of ontology concepts in text. The approach models the CR process as an information retrieval (IR) task by performing ad-hoc object retrieval over concept-token vector spaces built from the lexical representations of the concepts contained in the ontology. The concept-token vector spaces capture entity profiles, which are created from the overall token distribution, or more specifically, from their divergence from randomness [16] and mutual information [17]. The retrieval of candidates is then performed via an exact distance function.

The two proposed methods have been extensively evaluated against a standard baseline using the CRAFT corpus and ontologies. Experimental results show that, subject to the token distribution that emerges from a given ontology, both case sensitivity and information gain can be used as complementary strategies in generating alternative labels sets to improve the efficiency of the CR process.

The comparison against state of the art methods shows mixed results. While overall, ConceptMapper continues to outperform all existing methods, our approach is able to achieve a higher efficiency than Neji, NCBO Annotator and MetaMap on the Cell Ontology, GO Cellular Component and Sequence Ontology.

## Related Work

### Biomedical Concept Recognition Tasks

Several initiatives have been proposed over the years with the aim of harmonising NER and CR efforts, as well as working together towards manually annotated corpora that would support advances in the field. The two most prominent such initiatives are the BioCreative Challenges—the latest being BioCreative IV [18]—and the BioNLP Shared Tasks—the latest being the 2013 event [19]. Almost all tasks within these efforts target various forms of NER or event extraction, and only a very few have as main goal concept recognition. For instance, the Task B of the BioCreative IV GO Task aims to find GO concepts in plain text input, given a set of relevant genes, i.e., performing a form of context-driven CR, as opposed to plain CR that takes as input free text and a ontology with the aim of finding all mentions of the concepts defined by the respective ontology. The main reason behind the lack of CR tasks was, until recently, the lack of a manually annotated corpus to support a comprehensive comparison of proposed CR systems.

### Existing Concept Recognition Systems

Several CR systems have been developed and published over the course of the last ten years. In this section we provide a brief overview of the more widely known approaches by discussing their underlying processing mechanisms and possible advantages and limitations.

The **NCBO Annotator** [8] is a platform that enables biomedical concept recognition over unstructured text by exploiting over 200 ontologies published via the NCBO BioPortal [20]. The NCBO BioPortal includes many of the ontologies published by the Open Biomedical Ontologies Foundry [21], in addition to many others developed and submitted by specialised research groups, e.g., SNOMED-CT, NCI Thesaurus, International Classification of Diseases (ICD), Gene Ontology, Logic Observation Identifiers, Names and Codes (LOINC), Foundation Model of Anatomy, etc.

The NCBO Annotator operates in two stages: concept recognition and semantic expansion. Concept recognition is performed using Mgrep [22], which applies stemming as well as permutations of the word order combined with a radix-tree-search algorithm to allow for the identification of the best matches of dictionary entries to a particular text span. During semantic expansion, various rules such as transitive closure and semantic mapping using the UMLS Metathesaurus are used to suggest related concepts from within and across ontologies based on extant relationships. The mappings and the depth of transitive closure are customisable within the CR call.

The main advantage of the Annotator is the breadth of ontologies one can employ for CR purposes, in addition to its fast processing capabilities and its reliability. The deployment setting, however, does not allow the use of a non-public ontology, or in fact of any other ontologies except for those available in the NCBO BioPortal.

**MetaMap** [11] is a widely used system from the National Library of Medicine (NLM) for finding mentions of clinical terms based on CUI mappings to the UMLS Metathesaurus. The UMLS Metathesaurus forms the core of the UMLS and incorporates over 100 source vocabularies including the NCBI taxonomy, SNOMED CT or OMIM. MetaMap exploits a fusion of linguistic and statistical methods in a staged analysis pipeline. The first stages of processing perform mundane but important tasks such as sentence boundary detection, tokenization, acronym/abbreviation identification and POS tagging. In the next stages, candidate phrases are identified by dictionary lookup in the SPECIALIST lexicon [23] and shallow parsing using the SPECIALIST parser [24]. String matching then takes place on the UMLS Metathesaurus before candidates are mapped to the UMLS and compared for the amount of variation. A final stage of word sense disambiguation uses local contextual and domain-sensitive clues to arrive at the correct CUI.

MetaMap is highly configurable, for example, users have the option to specify their own vocabulary lists (e.g. for abbreviations), use negation detection and the degree of variation between text mention and UMLS terms. It is, however, also rigid in terms of ontologies used for concept recognition, since it has been developed strictly for UMLS. MetaMap is available as a downloadable software package.

**ConceptMapper** [14] is a generic CR tool developed within the UIMA [25] framework. It provides facilities for providing an arbitrary term dictionary, and has a range of parameters that control both how terms are processed (e.g., with stemming), and how terms are matched to text (e.g., via case-insensitive matching or with flexible word order). It has been demonstrated to achieve state of the art performance on the CRAFT corpus for a range of corpora, depending on what parameter settings are used [13].

**cTAKES** [10] from Mayo Clinic consists of a staged pipeline of modules that are both statistical and rule-based. The order of processing is somewhat similar to MetaMap and consists of the following stages: sentence boundary detection, tokenization, lexical normalisation (SPECIALIST lexical tools), part of speech (POS) tagging and shallow parsing trained in-domain on Mayo Clinic EHRs, concept recognition, negation detection using NegEx [26] and temporal status detection. Concept recognition is conducted within the boundaries of noun phrases using dictionary matching on a synonym-extended version of SNOMED CT and RxNORM [27] subset of UMLS. cTAKES was subject to a rigorous component-by-component evaluation during development. During this process, although the focus of testing was on EHRs, the system was also tested on combinations of the GENIA corpus of Medline abstracts. cTAKES is available as a desktop application.

**Whatizit** [28] is a modular infrastructure aimed at providing text mining services to the community. Each module has a specific functionality, such as named entity recognition or concept recognition. CR is available as a Web service and can be performed using publicly available ontologies and resources. The underlying process consists of a standard NLP pipeline augmented with a specific term matching algorithm that takes morphological variability into account.

**BeCAS** (the BioMedical Concept Annotation System) [29] is of the latest integrated CR systems. The pipeline of processes involves the following stages: sentence boundary detection, tokenization, lemmatization, part of speech tagging and chunking, abbreviation disambiguation, and CUI tagging. The first four stages are performed by GDep [30] a dependency parser that incorporates domain adaptation using unlabelled data from the target domain. CUI tagging is conducted using regular expressions for specific types such as anatomical entities and diseases. Dictionaries used as sources for the regular expressions include the UMLS, LexEBI [31] and the Jochem joint chemical dictionary [32]. During development the concept recognition system was tested on abstracts and full length scientific articles using an overlapping matching strategy. Concept recognition in BeCAS can only be performed on a predefined set of UMLS semantic types.

Finally, **Neji** [9]—a BeCAS successor—is an open source framework that delivers biomedical concept recognition in an automated and flexible manner. Neji’s processing pipeline includes built-in methods optimised for the biomedical domain and supports the application of both machine learning and dictionary-based approaches by automatically combining generated annotations and supporting concept ambiguity. Dictionary matching is realised via an efficient regular expression matching based on Deterministic Finite Automatons (DFAs). This is complemented with a list of non-informative words for the biomedical domain (to be ignored during the matching process) in order to cater for terms that are common English words. Machine Learning support is integrated via Gimli [33] (developed also by the authors of Neji), which employs Conditional Random Fields (CRFs) to identify various biomedical entity types.

Very few existing solutions attempt to take advantage of case sensitivity or of the distribution of the label tokens in the context of the underlying ontology. Some approaches, in particular for the BioCreative Challenges and the BioNLP Shared Tasks, exploit case sensitivity (e.g., [34]). ConceptMapper can be configured to exploit it, but only in an on-or-off manner. Others, apply IR techniques, such as query language models or standard ranking functions like Okapi-BM25 that can make use of case (in)sensitive matching—see [35], [36] or [37] (note that, except for ConceptMapper, all these examples target NER and not CR). To date, no solution proposes a framework to detect case-sensitivity.

From a methodological perspective, the work of Gaudan et al. [38] is the closest to our information gain framework. The authors investigate and evaluate—addressing the distinct task of sentence- or paragraph-level annotation of GO terms rather than bounded mention detection—the evidence for and specificity of a term, as well as the proximity of multiple terms using information theoretic metrics—See also the work of Verspoor *et al* [15] exploring term mapping into the GO structure. Such an approach would, in practice, complement the information gain with a measure of specificity, computed based the distribution of the term in the underlying ontological hierarchy. We intend to study this complementarity in our future work.

## Background

### Divergence from randomness

Divergence from randomness (DFR) [16] is a method that emerged as a generalisation of one of the early information retrieval models—Harter’s 2-Poisson indexing model [39]. The 2-Poisson model relies on the premise that informative words are supported by an *elite set* of documents, in which these words tend to be more frequent in comparison to the rest of the documents. Moreover, there are also words that are not supported by such an elite set, and hence their frequency follows a random distribution. Harter’s model has been explored thoroughly in the IR domain and has led, among other outcomes, to the well-known BM25 ranking function.

In general, a DFR model relies on the assumption that a word carries more information within a particular document if it has a larger divergence of the within-document frequency from its frequency in the collection. Consequently, the weight of the words is inversely related to the probability of frequency within a document *d*, using a particular model of randomness *M* (see Eq. 1).

$$\begin{array}{c} weight\left(t|d\right)=-log\;Prob_{M}\;\left(t\in d|Collection\right) \end{array}$$ (1)

A widely used model of randomness has been the binomial distribution, as defined in Eq. 2.

Prob(t∈d|Collection)=TFtf*ptf*qTF-tf (2)

where *TF* is the frequency of *t* in the *Collection*, *tf* is the frequency of *t* in document *d*, *N* is the total number of documents in the *Collection*, *p* = 1/*N* and *q* = 1 − *p*. The full weight of *t* associates a high frequency word to a low risk of the word not being informative, but also to a small information gain. Hence, in order to find the information gain of *t*, one needs to consider only the fraction of the full weight that is associated with the risk probability—see Eq. 3, where *P*_*risk*_ is 1 − *Prob*(*t* ∈ *d*∣*d* ∈ *Elite set*).

$$\begin{array}{c} gain\left(t|d\right)=P_{risk}*\left(-log\;Prob\left(t\in d|Collection\right)\right) \end{array}$$ (3)

Hence, the more often the word occurs in the elite set, the less its frequency is due to randomness. A common way of quantifying *P*_*risk*_ is based on the ratio of two Bernoulli processes (Eq. 4).

$$\begin{array}{c} P_{risk}=\frac{TF}{df*(tf+1)} \end{array}$$ (4)

with *df* = the number of documents containing the word.

Finally, in order to take into account the length of the containing documents, the word frequency can be normalised via a standard normalised document length, as in Eq. 5.

$$\begin{array}{c} tfn=tf*log\left(1+\frac{sl}{dl}\right) \end{array}$$ (5)

where *dl* is the document length containing *t* and *sl* is the standard normalised document length. Eq. 6 provides the complete formulation of the gain model.

gain(t|d)=-logTFtfn*ptfn*qTF-tfn*TFdf*(tfn+1) (6)

This framework will be applied in our context by using the ontology as a collection of concepts (which denote the documents in the DFR framework) and by analysing the information gain brought by tokens within the lexical groundings of the ontological concepts (i.e., labels and synonyms).

### Mutual information

Mutual information (MI) denotes a measure of information overlap between two random variables [17]. It is defined as in Eq. 7.

$$\begin{array}{c} I\left(X;Y\right)=\sum_{x,y}p\left(x,y\right)ln\frac{p(x,y)}{p(x)p(y)} \end{array}$$ (7)

where *p*(*x*) and *p*(*y*) represent the marginal probabilities of the random variables *X* and *Y* and *p*(*x*, *y*) is the joint probability. Mutual information (or the information overlap) is 0 when *X* and *Y* are independent, i.e., *p*(*x*)*p*(*y*) = *p*(*x*, *y*). Using MI as a starting point, several other measures have been proposed, such as pointwise mutual information (PMI), which quantifies the divergence between the actual joint probability of two events and the expected probability of the individual events under the assumption of independence. It is easier to interpret MI as measures of independence (i.e., closeness to 0) than as concrete measures of correlation, due to the lack of lower and upper bounds. Nevertheless, MI has been successfully used in various models in information retrieval or text mining (either directly or normalised), in particular as measures of co-occurrence or collocation between terms, based on the intuition that the higher the MI, the more correlated two events are.

## Materials and methods

### Method overview

A typical concept recognition task consists of three phases: (i) concept processing—on the ontology side; (ii) candidate generation—using the provided input; and (iii) candidate matching—i.e., finding the most relevant concepts for a given candidate. The concept processing phase ranges in complexity from basic (i.e., application of a typical NLP pipeline on concept labels and synonyms) to advanced, e.g., creating token distributions within and across ontologies or capturing token context. Most of the existing approaches share, to a large extent, the candidate generation phase, typically relying on some form of shallow (or deep) natural language processing. Approaches vary in how they perform concept processing and candidate matching. The matching strategy, on the other hand, depends on the underlying data representation model and on the desired matching goal. A quantification of the exact or overlapping matching between candidates and processed concepts can be achieved either directly (i.e., 1-to-1 comparison) or via diverse similarity metrics.

**Fig 1:**
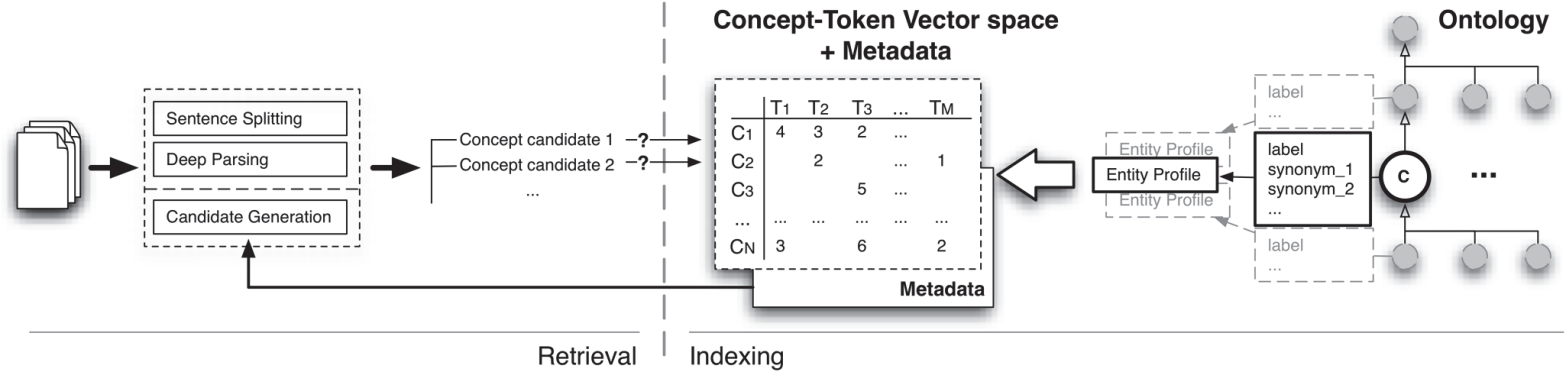
General overview of our proposed concept recognition approach. Similar to a standard Information Retrieval system, the framework consists of two phases: indexing and querying. The indexing step uses entity profiles, generated from the set of labels and synonyms associated with each oncological concept, to create concept-token vector spaces. The entity profiles are also used in assessing case sensitivity and generating alternative labels based on token information gain and mutual information. The querying step performs standard pre-processing operations, in addition to using a custom ranking function that leads to retrieving concepts based on exact boundary matching.

Fig. 1 depicts the general overview of our proposed approach. This follows the same pattern as discussed above, and it is split into two major steps: indexing and retrieval. Indexing is realised in a ontology-specific context, i.e., it is performed individually for each provided ontology without considering or compiling cross-ontology aggregated information. Firstly, the need for case sensitivity is ascertained, followed by a process of building entity profiles from individual ontological concepts, and finally by the creation of a concept-token vector space. Case sensitivity, in addition to other statistics, are retained as part of the index metadata and used in the retrieval process. The retrieval step performs the standard candidate generation operations (although taking case sensitivity into account when required) followed by ad-hoc object querying on the concept-token vector space using a custom ranking function. Concrete details of these steps are provided in the following sections.

### Determining case sensitivity

**Fig 2:**
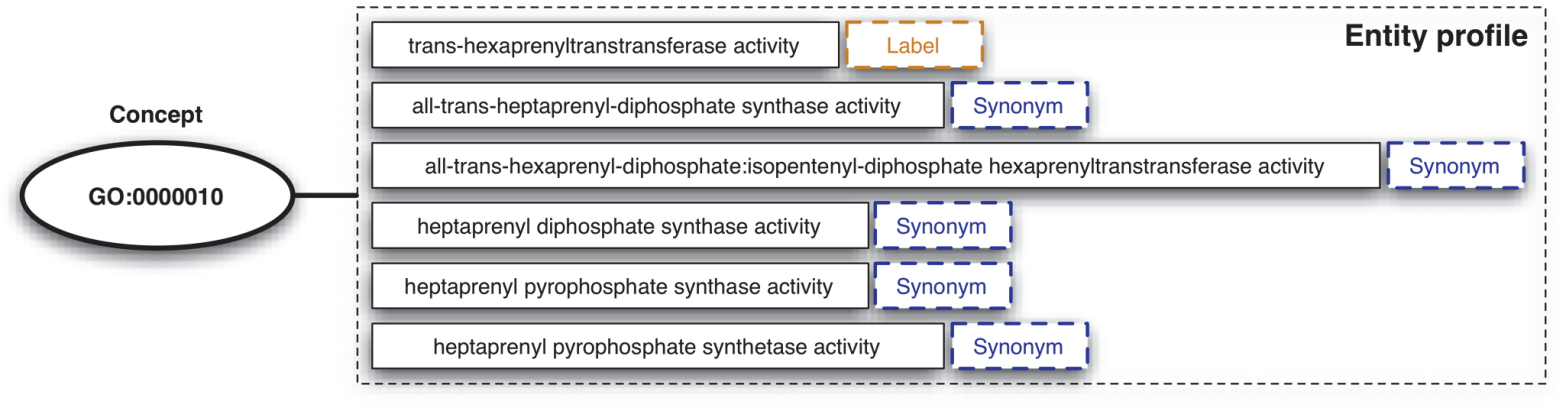
Entity profile example for concept GO:0000010. The entity profile aggregates the set of labels (preferred and alternative) and the synonyms defined by the concept under scrutiny. In this example, the concept has only one label, i.e., *trans-hexaprenyltranstransferase activity*, and five synonyms.

Case sensitivity is determined at the global, ontology level. We create so-called *entity profiles*, one per ontological concept, by aggregating the set of labels (preferred and alternative) and synonyms defined in the context of the concept under scrutiny. Fig. 2 depicts the entity profile for the concept GO:0000010 (*trans-hexaprenyltranstransferase activity*). This consists of one label and five synonyms.

A concept-oriented distribution of the case sensitive tokens is built from its underlying entity profile—i.e., the distribution of case sensitive tokens within the set of all tokens present in the labels and synonyms of a particular concept. In our study, *case sensitive tokens* are characterised by: (i) a camel case shape, e.g., *MetaMap*; (ii) an all upper case form, e.g., *STAR* or (iii) *FGFR3*; or capitalisation mixed with presence of digits and / or symbols, e.g., *Tyr336*. Given the above definition, we consider an ontological concept to be case sensitive if its underlying token distribution (defined by the entity profile) contains at least one case sensitive token.

The aim is to capture the frequency and uniformity of such case sensitive concepts, in order to understand the general character of the ontology. These concepts are deemed important if they are both frequently present as well as uniformly distributed across the set of all concepts within an ontology. Consequently, in computing the ontology case sensitivity status (CSS), we use a function that comprises two components, as shown in Eq. 8.

$$\begin{array}{c} CSS=\frac{1}{2}*\left(\left(e^{-{\text{Δ}}_{CS}}-\frac{{\text{Δ}}_{CS}}{e}\right)+\frac{Freq_{CS}}{N_{C}}\right) \end{array}$$ (8)

where, *N*_*C*_ is the total number of concepts in the ontology, *Freq*_*CS*_ is the number of case sensitive concepts and Δ_*CS*_ is the standard deviation of the window difference between two consecutive case sensitive concepts. Δ_*CS*_ is defined in Eq. 9.

$$\begin{array}{c} {\text{Δ}}_{CS}=\sqrt{\sigma_{C}^{2}};\sigma_{C}^{2}=\frac{1}{N_{C}}*\sum_{1}^{N_{C}}{\left(W_{i}-Avg\left(W_{i}\right)\right)}^{2} \end{array}$$ (9)

$$\begin{array}{c} Avg\left(W_{i}\right)=\frac{1}{N_{CS}}*\sum_{1}^{N_{CS}}W_{i} \end{array}$$ (10)

*Avg*(*W*_*i*_) is defined in Eq. 10, *N*_*CS*_ is the total number of case sensitive concepts and *W*_*i*_ is the window difference between two consecutive case sensitive concepts, or the number of non-case sensitive concepts between two case sensitive concepts.

Δ_*CS*_ shows the extent to which the appearance of a case sensitive concept deviates from a uniform distribution. A standard deviation of 0 represents a perfectly distributed appearance. Consequently, we have introduced a decreasing exponential that increases the value of the uniformity factor inversely proportional to the decrease of the standard deviation—i.e., the lower the standard deviation, the higher the uniformity factor. The second component of *CSS* captures the fraction of case sensitive concepts in the ontology—the larger the fraction, the more probable is the necessity for case sensitive processing. As can be observed, *CSS* is undefined for *Freq*_*CS*_ = 0 (i.e., ontologies with no case sensitive concepts) and reaches a maximum of 1 when all concepts in an ontology are case sensitive. Hence, positive values, as well as negative values that are in close proximity of 0 are good indicators of an ontology that requires case sensitive processing.

In order to achieve a better understanding of a threshold for *CSS* we performed an experiment. We collected 50 random ontologies from the NCBO BioPortal and computed their corresponding *CSS* value. Using these results we were able to observe two fairly well formed clusters of values: values less than −3.75 and values larger than −1. A manual inspection of the ontologies led to the expected conclusion that ontologies associated with *CSS* values in the second group (i.e., larger than −1) were dominated by case sensitive concepts. Consequently, we have used this threshold throughout our experiments reported in Section.

### Entity profile creation

As defined above, an entity profile aggregates the set of all lexical representations of an ontological concept (as defined in a given ontology). This includes all types of labels (i.e., basic, preferred or alternative) and synonyms. The processing steps required to build entity profiles depend on the case sensitivity status.

In the case of a **case insensitive** ontology, we perform standard shallow NLP on the lexical representations, i.e., tokenization, POS tagging, lemmatisation and lexical normalisation. Tokens that contain special characters (e.g., “-”) are divided into multiple terms. For example, “*C3/C5 convertase complex*” is transformed to “*C3 C5 convertase complex*”. Finally, we apply post-processing operations to remove determiners and prepositions and to lower case all tokens. From an implementation perspective, we have used the GENIA Tagger [40] for tagging and lemmatisation.

A **case sensitive** ontology is processed in a slightly different manner, i.e., we distinguish plain tokens from case sensitive tokens (based on their shape—see previous section). Plain tokens are processed as above, while case sensitive tokens are not divided, lemmatised and lower cased—i.e., we maintain their original form.

The final step in this entity profile creation is understanding the information gain of each token in the context of a lexical representation by studying their divergence from randomness and mutual information with other tokens.

We believe that the DFR hypothesis (as defined in the Background section) is valid, at a smaller scale, within an ontology. More precisely, there exist tokens that are supported by an *elite set of entity profiles* and are the most informative in the context of their underlying concepts, as well as tokens that do not bring an information gain. This assumption is supported by the uniform manner in which ontological concepts are defined. For example, the large majority of classes in GO Cellular Component define “*complexes*”—e.g., “*Complement component C1 complex*” or “*General secretion pathway-associated complex*”. Similarly, the Protein Ontology defines “*proteins*” (e.g., “*Myc protein*” or “*Noggin protein*”), while GO Biological Process and Molecular Function defines “*activities*” (“*alpha-1, 6-mannosyltransferase activity*” or “*adenine aminase activity*”). In practice, however, in scientific publications or clinical reports, these concepts are usually referred to by their “*short*” name, e.g., “*adenine aminase*” or “*Myc*”, which has a negative impact on the CR process. Our goal is to find these tokens and produce alternative lexical representations that omit them.

We, hence, apply the DFR model introduced in the previous section using the underlying ontology as a *Collection* of documents—i.e., entity profiles. Taking into account our context, the elements of the DFR equations have been interpreted as follows: (i) *d*—an entity profile; (ii) *t*—a token present in the entity profile (e.g., “*activity*”); (iii) *N*—the total number of entity profiles (or concepts) in the ontology; (iv) *TF*—the frequency of *t* in *N*; (v) *tf*—the frequency of *t* in a given entity profile; and (vi) *df* is the number of entity profiles containing *t*. Finally, *dl* and *sl* in Eq. 5 have been computed by averaging the length of the lexical representations within an entity profile (*dl*), and by averaging the length of all lexical representations in the ontology (*sl*), respectively.

Given a concept label (“*adenine aminase activity*”), the DFR process results in an information gain (IG) associated with each token of this label. This IG can then be used to filter out low-valued tokens and create alternative labels that omit them. The filtering process is, however, challenging. A standard solution could be to set a global threshold over IG and removing all those terms that do not satisfy it. Unfortunately, such a solution is very rigid (it does not take into account the local, lexical representation, context) and would lead to the generation of an important number of false positives.

There are three representative scenarios that need to be considered when filtering low information gain terms:

1. Multi-token lexical representations with low IG tokens that should be omitted—e.g., “*activity*” in GO:0000034 (*adenine aminase activity*), resulting in “*adenine aminase*”;
2. Multi-token lexical representations with low IG tokens that should be retained—e.g., “*cell*” in GO:0033655 (*host cell cytoplasm part*); and
3. Bigrams with or without low IG tokens, such as CL:0000210 (*photoreceptor cell*) in Cell Ontology—where “*cell*” is a low IG token and we would be interested in removing it, or GO:0010467 (*gene expression*) in Gene Ontology—where neither token has a low IG, yet encountering *expression* should lead to an association with the concept.

Below, we discuss our solution for addressing these scenarios.

#### Processing multi-token lexical representations

The decision to omit or retain low IG tokens in multi-term lexical representations depends on two aspects: (i) detecting outliers in the set of tokens of the lexical representation, and (ii) understanding the relationship of these outliers with respect to the neighbouring tokens. In order to detect outliers we use basic statistical measures. Given a particular multi-token lexical representation, we compute the mean and standard deviation (SD) of the information gain of all tokens and we mark as outliers all tokens that have an IG less than one standard deviation away from the mean. For example, the information gain associated with the tokens of the label “*adenine aminase activity*” (GO:0000034) is: {2.5606, 4.2291, 0.3816}, which yields a mean of 2.3905 and a standard deviation of 1.5753. As a result, the one standard deviation away from the mean threshold is set at 0.8151 (i.e., 2.3905 – 1.5753) and the “*activity*” token is marked as an outlier. The same computation for the concept GO:0033655 (*host cell cytoplasm part*) leads to “*cell*” being marked as an outlier (with an information gain of 0.0715).

Intuitively, in the examples above, the “*activity*” token should probably be discarded, while the token “*cell*” should be retained—discarding the latter would produce a token that has no real domain semantics. Consequently, the second aspect is required to gain a better understanding of the importance of each token, and mutual information provides an appropriate framework to study this.

Mutual information quantifies the degree of information overlap between two variables and has been widely used as a measure of correlation (or independence) between words. The pairwise MI of all possible bigrams in the lexical representations in an ontology displays a quasi-normal distribution (see Supplementary Information S1–S6). The large majority of the pairwise MI values are in the close proximity of 0 (i.e., independence), while highly correlated bigrams form the tails of the distribution. This is to a large extent natural (at least in the biomedical domain) because the large majority of token pairs would be very rarely used in multiple concept definitions and only a few would be re-used several times—e.g., “*protein complex*” in the Protein Ontology: over 200 occurrences.

We can, hence, use MI to verify the correlation between the tokens marked as outliers and their neighbours. Tokens that are highly correlated with at least one of the adjacent tokens (i.e., forming a highly correlated bigram) should be retained, while those that are not correlated—i.e., close to being independent—should be discarded. In order to achieve this, we use the distribution of the pairwise MI values computed over the entire ontology and build an independence interval (II) to quantify the degree of correlation / independence via the interquartile range rule—as per Eq. 11

$$\begin{array}{c} \text{II}=\left[m*Q_{1}-n*IQR\right]:\left[m*Q_{3}+n*IQR\right] \end{array}$$ (11)

where *m* and *n* are constants used to adjust the independence interval, *Q*_1_ is the first quartile of the pairwise MI distribution, *Q*_3_ is the third quartile of the distribution, and *IQR* = *Q*_3_ − *Q*_1_ is the interquartile range. Values outside the independence interval denote correlated bigrams, since they represent the tails of the distribution, while values inside the interval denote independence. The constants *m* and *n* enable us to move the thresholds of the independence interval subject to the underlying distribution, which has unique characteristics for each ontology.

#### Processing bigrams

Bigrams are a special case of multi-token lexical representation where the rules listed above cannot be applied—two IG values will always be within one SD from their mean. Furthermore, in addition to discarding low IG terms, we may be interested in splitting also labels that do not necessarily contain such tokens—see the “*gene expression*” example above. In both cases, the MI value of the bigram is important.

To solve this challenge, we revert to the initial goal of detecting outliers—i.e., tokens with low IG values. However, in a bigram context, instead of relying of the local comparisons (i.e., within the lexical representation), we approach the challenge from a global perspective, and follow similar steps: (i) compute a global IG mean value for each token—using the values from multi-token lexical representations and bigrams; (ii) calculate the overall mean and standard deviation of all tokens; (iii) use *Mean*_*IG*_ − *k* * *SD*_*IG*_ as upper threshold for detecting outliers—where *k* is a constant which depends on the underlying distribution of the information gain and *SD*_*IG*_ is the standard deviation of the information gain.

This enables us to create a list of global low IG tokens—e.g., “*cell*” in Cell Ontology. For each bigram containing such tokens we then verify the MI—as discussed above. Those that are within the independence interval are split, with the more informative token being retained. For example, in the case of CL:0000210 (*photoreceptor cell*) in Cell Ontology, this will lead to generating an alternative label using only the token “*photoreceptor*”.

The second challenge—splitting lexical representations without low IG tokens—is addressed by considering the global MI of the tokens and their position in the bigram (i.e., on the left or the right of the bigram). A bigram is represented by the pair (*LT*, *RT*), where *LT* is the left token and *RT* is the right token in the bigram. For example, the bigram *gene expression* is represented by *LT* = *gene* and *RT* = *expression*. Subject to the token correlation status (i.e., outside or inside the independence interval), the decision takes into account two aspects computed both in balance between the tokens, as well as in the individual context of the token:

- the global MI of the token associated with its current position in the bigram—e.g., the global MI of *expression* in the previous bigram example computed using all bigrams where *expression* is *RT*. We represent this value using the notation: *MI*(*RT* ⇝ *POS*_*R*_), where *POS*_*R*_ is the position on the right in the bigram
- the global MI of the token associated with the opposite position in the bigram—e.g., the global MI of *expression* computed using all bigrams where *expression* is *LT*. This value is represented using the notation: *MI*(*RT* ⇝ *POS*_*L*_), where *POS*_*L*_ is the position of the left in the bigram

The global MI for a given position is defined in Eq. 12 and represents the average MI value for all bigrams containing a given token in the given position—e.g., as mentioned above, all bigrams where *expression* is the token on the right.

$$\begin{array}{c} MI\left(T⇝POS\right)=\frac{1}{n}\sum_{i=1}^{n}MI\left(T,t_{i}\right)|T\in \left\{LT,RT\right\};POS\in \left\{POS_{L},POS_{R}\right\} \end{array}$$ (12)

If the bigram tokens are correlated, we are interested in retaining the token that shows an overall higher information content given its position in the bigram, or more concretely: 1. a higher global MI associated with its current position in comparison with the opposite position; and 2. a lower global MI associated with the opposite position, in comparison with the same value for the other token in the bigram. This information is captured using Eq. 13 and 14. Firstly, we define a sign function (Eq. 13) to determine the token associated with a lower global MI for the position opposite to its current position in the bigram, i.e., the difference between *MI*(*LT* ⇝ *POS*_*R*_) and *MI*(*RT* ⇝ *POS*_*L*_).

$$\begin{array}{c} S=sign(MI\left(LT⇝POS_{R}\right)-MI\left(RT⇝POS_{L}\right)) \end{array}$$ (13)

Secondly, we asses the ratio between the global MI associated with the opposite position and the global MI associated with the current position in the context of both tokens, as defined in Eq. 14. A subunitary ratio denotes a stronger information content associated with the current position, while an overunitary ration denotes the opposite. The sign function has the role to shift the focus from one token to the other.

$$\begin{array}{c} S*\left[{\left(\frac{MI(T_{L}⇝POS_{L})}{MI(T_{L}⇝POS_{R})}\right)}^{S}*{\left(\frac{MI(T_{R}⇝POS_{L})}{MI(T_{R}⇝POS_{R})}\right)}^{S}\right] \end{array}$$ (14)

The above listed conditions translate into the following interpretations of the results for Eq. 14: (i) a positive subunitary value leads to the second token of the bigram being retained; (ii) a negative subunitary value leads to the first token being retained; (iii) any other values do not split the lexical representation—including the values for which the equation is not defined, i.e., *MI*(*T*_*L*_ ⇝ *POS*_*R*_) = 0 and *MI*(*T*_*R*_ ⇝ *POS*_*R*_) = 0.

A similar aim is targeted also for uncorrelated tokens, but without taking into account the balance in positions. Consequently, we interpret the resulting values of Eq. 15 in the following manner: (i) a positive subunitary value leads to the second token of the bigram being retained; (ii) a negative overunitary value leads to the first token being retained; (iii) any other values are discarded including those for which the equation is not defined, i.e., *MI*(*T*_*R*_ ⇝ *POS*_*L*_) = 0.

$$\begin{array}{c} S*\frac{MI(T_{L}⇝POS_{R})}{MI(T_{R}⇝POS_{L})} \end{array}$$ (15)

To have a better understanding of the proposed mechanism, we describe the “*gene expression*” example from GO_BPMF, starting from *MI*(*gene*, *expression*) = 0.00061, which shows that the tokens are almost independent. The values for position dependent MI in the bigram are the following:

- *MI*(*gene* ⇝ *POS*_*L*_) = 1.066;
- *MI*(*gene* ⇝ *POS*_*R*_) = 1.4;
- *MI*(*expression* ⇝ *POS*_*L*_) = 0.19;
- *MI*(*expression* ⇝ *POS*_*R*_) = 0.5.

This leads to *S* = *sign*(*MI*(*gene* ⇝ *POS*_*R*_) − *MI*(*expression* ⇝ *POS*_*L*_)) = *sign*(1.4 − 0.19) = +1, and to the result shown in Eq. 16. Consequently, an alternative label will be generated using the token “*expression*”.

$$\begin{array}{c} +1*\left[{\left(\frac{1.066}{1.4}\right)}^{+1}*{\left(\frac{0.19}{0.5}\right)}^{+1}\right]=0.0723 \end{array}$$ (16)

### Concept-Token vector space

The last element of the indexing phase is representing the lexical groundings of the ontological concepts in a way that allows a fast and efficient retrieval. We adopt the vector space model to capture the association between concepts and tokens, where columns denote tokens and rows lexical representations or alternative labels. However, instead of using the standard way of marking the presence of a token in a lexical representation (i.e., 1 / 0), we encode the relative placement of the term in the lexical representation—similar to building Hypespace Analogue to Language (HAL) spaces [41]. More concretely, for each token we record the value of (*L* − *i* + 1), where *L* is the length of the label and *i* is the index of the token in the list of tokens denoting the label (using a 1-based indexing—i.e., the first index in the list is 1). While not used at full potential in the experiments discussed later in the paper, subject to the ranking function, this representation enables directly (without additional processing) both exact and nested matching, as well as proximity-based queries.

### Candidate generation and retrieval

The retrieval step generates candidate queries using the text processing steps employed also by the entity profile creation phase—i.e., subject to case sensitivity, a given input is tokenized, tagged and lemmatised, followed by the post-processing operations of removing determiners and prepositions. Given a query *Q* (input candidate), the highest ranked concept candidates are retrieved using the score function listed in Eq. 17.

$$Score(Q,C)=\frac{Q_{N}-Q_{NF}}{Q_{N}}*\frac{\sum_{i\;=\;1}^{Q_{N}}C_{q_{i}}}{\sum_{i\;=\;1}^{C_{N}}C_{t_{i}}}$$ (17)

where *Q*_*N*_ is the query length in tokens, *Q*_*NF*_ is the number of query tokens inexistent in the concept-token vector space, *C*_*N*_ is the token length of a lexical representation of concept *C*, *C*_*q*_*i*__ is the distance value of *q*_*i*_ in the lexical representation of concept *C* and *C*_*t*_*i*__ is the distance value of token *t*_*i*_ in the lexical representation of concept *C*. This general form of the scoring function can be used for nested and exact matching. However, our aim is to perform exact matching, which translates into finding the concept *C* for which *Q*_*NF*_ is 0 and the sum over the distances of the query tokens is the same as the sum over the distances of the tokens composing the lexical representation of *C*—i.e., solving *Score*(*Q*, *C*) = 1.

## Experimental results

### Experimental setup

We have performed an extensive series of experiments to evaluate our approach using the CRAFT corpus [12]. The CRAFT corpus is the first comprehensive resource that enables gold standard-based concept recognition evaluation. The public 1.0 version consists of 67 full-length articles that have been manually annotated against several ontologies covering various aspects, such as proteins, chemical entities or cells. We refer the reader to [12] for the corpus description. In our experiments we have used the following ontologies from CRAFT: Cell Ontology (CL), GO Cellular Component (GO_CC), GO Biological Process and Molecular Function (GO_BPMF), ChEBI, Protein Ontology (PRO) and Sequence Ontology (SO).

Our experimental setup has been the following:

1. A baseline has been created by indexing the 6 above-listed ontologies using the basic framework, without case sensitivity assessment and information gain.
2. In parallel, the ontologies have also been indexed using only case sensitivity assessment, only the information gain framework and both. In order to gain a better understanding of the IG framework, we have used different combinations of parameters for *m* and *n*, to vary the independence interval (see Eq. 11), and for *k*, to vary the outlier threshold;
3. Each resulting index (baseline, with case sensitivity, with information gain and with both) has been used to annotate the 67 CRAFT publications. Precision, Recall and F1 have been computed to record the concept recognition efficiency.

Our experiments focus on exact matching—i.e., finding the exact boundaries of an entity of interest and associating it to the corresponding ontological concept.

**Fig 3:**
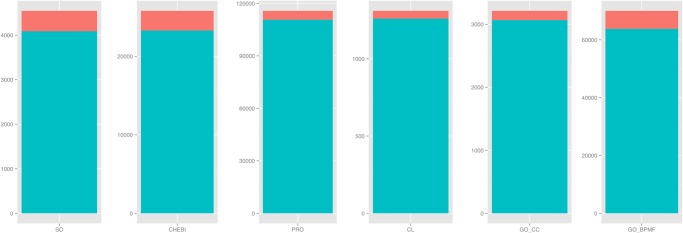
The effect of using the information gain framework on the size of textual groundings defined in the six CRAFT ontologies. The number of additional textual groundings introduced via IG is not proportional to the original number of defined labels or synonyms, but rather depends on the number of low information gain terms. The percentage of these additional groundings ranged from 3.96% in CL to 11.20% in SO.

For completeness purposes, here we list the outcome of the case sensitivity assessment: (i) CL—FALSE; (ii) GO_CC-TRUE; (iii) GO_BPMF - TRUE; (iv) ChEBI-TRUE; (v) PRO-TRUE; (vi) SO-TRUE; The results achieved by the best combination of parameters are discussed in the following section. These were recorded for the following parameter values: (i) *k* = 3 for all ontologies; (ii) *m* = 0.8, *n* = 0, for CL; (iii) *m* = 0.9, *n* = 0, for GO_CC; (iv) *m* = 1, *n* = 0.1, for GO_BPMF; (v) *m* = 0.9, *n* = 0, for ChEBI; (vi) *m* = 1, *n* = 0.2, for PRO; (vii) *m* = 0.7, *n* = 0, for SO. A view over the behaviour of the efficiency measures with various values for these parameters can be found in the Supplementary Information S7 to S12. Fig. 3 depicts the effect of the information gain framework on the size of textual groundings defined in the CRAFT ontologies. The increase in number of labels defined ranged from 3.96% in CL to 11.20% in SO. GO_BPMF recorded an increase of 9.74%, while CHEBI one of 10.74%.

### Results

Table 1 lists the results achieved by our approach in various configurations. From a case sensitivity perspective, the results are divided: with the exception of the Cell Ontology, which has been assessed as non-case sensitive (and hence the results are the same), GO_CC and PRO have recorded an increase in F-Score when compared to the baseline (2.05% and 7.15%), while most of the other ontologies have been affected negatively (in both cases the decrease was less than 1% F-Score). It is interesting to observe though, that case sensitivity produced positive results by having two different behaviours. In the case of GO_CC, it filtered out some of the false positives generated from the original input and which were deemed as case sensitive candidates—i.e., candidates that were incorrect independently on the case sensitivity status, but which were eliminated in the matching process because they were case sensitive and the ontology contained them in a lower cased version. In PRO, on the other hand, the process was able to take advantage of the definition of case sensitivity and has disambiguated terms based on their shape—for example, PR:000013884 (*AGE*) was no longer associated with the common English word “*age*” or the process “*ageing*”, both consistently present in the input documents. Finally, GO_BPMF was not affected by case sensitivity, although its initial case sensitivity assessment has been positive, because the case sensitive terms do not resemble common English words, as in the case of PRO (e.g., GO:0008130—*PMNL collagenase activity*).

**Table 1:** Experimental results: Baseline vs. Case sensitive vs. Information gain.

Ontology	Baseline			Case sensitive (CS)			Information Gain (IG)			CS + IG		
	P	R	F1	P	R	F1	P	R	F1	P	R	F1
CL	84.95	72.22	**78.07**	84.95	72.22	**78.07**	83.02	71.67	76.93	83.02	71.67	76.93
GO_CC	80.29	71.70	75.75	83.32	72.95	**77.79**	80.93	73.19	**76.86**	83.56	73.18	**78.02**
GO_BPMF	55.48	23.55	33.06	55.48	23.55	33.06	47.67	34.04	**39.72**	47.67	34.04	**39.72**
ChEBI	47.83	54.54	**50.97**	47.34	53.28	50.13	44.35	51.97	47.86	43.83	50.82	47.06
PRO	28.59	63.53	39.43	41.72	52.72	**46.58**	27.48	63.96	38.45	40.39	53.33	**45.96**
SO	50.68	52.39	**51.52**	50.50	51.76	51.12	45.60	50.43	47.89	43.71	48.73	46.08

Bolded values denote an increase in F-Score against the baseline. Using a case-sensitive processing approach leads to an increase in F-Score ranging from 0.02 to 0.07 on three of the six ontologies—i.e., CL, GO_CC and PRO. Similarly, information gain improves the F-Score in two ontologies—i.e., GO_CC and GO_BPMF. The efficiency of the combined approaches mirrors proportionally their individual behaviour.

Information gain had a similar behaviour. It resulted in an increased F-Score in two ontologies (GO_CC and GO_BPMF—1.11% and 6.66%) and had a negative effect on the rest (due to over-generation of alternative labels representing false positives). In GO_CC and GO_BPMF, the framework found the terms “*complex*” and “*regulation*” + “*activity*”, respectively, as outliers, while in CL and PRO, it found the terms “*cell*” and “*protein*”, respectively. In the first case, this led to a positive outcome (e.g., GO:0004189—*tyrosinotubulin carboxypeptidase activity* or *TTCPase activity*—had generated alternatives in *tyrosinotubulin carboxypeptidase* or *TTCPase*). In the second case, the outliers did not have a direct impact on the results, but rather the mutual information between some terms, which led to confusion, mostly because of the ambiguous nature of the terms that were retained in the filtering process (e.g., PR:000004689—*cellular-E10* had a generated alternative in *E10*, which resulted in a series of false positives).

Finally, in the third part of Table 1 we can observe a cumulative effect when combining case sensitivity and information gain—i.e., the results are almost proportionally lower or higher depending on the individual effect of each component. For example, although PRO had an important increase in F-Score due to case sensitivity (46.58% from 39.43% in the baseline), it achieved a lower F-Score in the last column because information gain had a negative effect (45.96% vs. 46.58%). Similarly, in GO_CC the positive effect of each of the two elements has led to an increased overall F-Score in their combined version (78.02% from 77.79% case sensitivity and 76.86% information gain).

## Discussion

### Error analysis

As shown above case sensitivity and token IG improve the CR accuracy when compared against a standard baseline. On the other hand, the improvement is not uniform and, subject to the underlying ontology, our methods may lead to less satisfactory results. There are three types of errors we have encountered when analysing the experimental results, each of which is discussed below.

#### General errors

Most of these errors are related to the inability of finding a concept candidate for a text span and lead to a lower recall. They are present in the baseline and then carried throughout the additional processing steps. The underlying issues are related to: (i) lack of synonyms—text spans referring to certain concepts via synonyms that are not captured in the ontology, e.g., “*biogenesis*” referring to GO:0022607 (*cell structure assembly*) in GO_BPMF; (ii) lack of inference—text spans denoting concepts more generic than those with which are annotated, e.g., “*antibody*” referring to GO:0019814 (*immunoglobulin complex*) in GO_CC; and (iii) lexical representations that contain gaps, e.g., “*cells in … epithelial … regions*” referring to CL:0000066 (*epithelial cell*) in Cell Ontology; this issue emerges from the inability of our input processing pipeline to support gaps in text spans.

#### Errors due to case sensitivity

We have initially believed that case sensitive could not affect negatively the CR process, based on the assumption that concepts follow a uniform case sensitive notation—i.e., the shape of the concept labels used in ontologies is the same with that used in the literature. This assumption has, however, failed and has led to a decrease in F-Score in two ontologies: ChEBI and SO. A closer look at the results has revealed terms defined in the ontologies in a case insensitive manner and used in the input differently. For example, CHEBI:49168 is defined as “*dopa*” and encountered in the input as “*DOPA*”. Similarly, SO:0001457 is defined as “*unigene cluster*” and referred to in the input as “*UniGene cluster*”—please note that the term “*UniGene*” conforms to our definition of case sensitive term.

#### Errors due to information gain

These errors are related to the over-generation of alternative lexical representations, which leads to an increased number of false positives. For example, using our framework with diverse independence intervals for mutual information may lead to alternative lexical representations for the concept PR:000023929 (*secretion monitor*), i.e., “*monitor*”.

Overall, the results show that the proposed framework is able to improve the efficiency of the CR process in ontology-specific contexts, subject to the distribution of the tokens defined by the underlying ontology. There are several ways in which both components of the framework can be employed within the context of an existing CR method. For example, given an ontology of interest, if annotated data is available for the target domain, a decision on their inclusion in the CR process can be reached using an evaluation strategy similar to the we have described in the previous section. If annotated data is not available, the indirect outcomes of the two components provide an alternative for reaching a decision. In the case of the case sensitivity assessment, the CSS value can be inspected to study its proximity to the threshold value we have proposed. Similarly, the information gain framework can be used to generate low IG candidates for different independence intervals. These candidates could then be included in the generation of alternative labels. One of the advantages of the IG method we have proposed is its structure—subject to the underlying token distribution, one may decide to use the entire framework (covering the three scenarios we have discussed), or only specific components, such as the multi-token lexical representation scenario that excludes bigrams. Furthermore, the framework can be augmented with a distributional semantics model to refine both IG and the MI values. Finally, our method can be easily transformed into a (semi-) supervised learning approach, in which the independence interval could be learned with the help of a gold standard like CRAFT. Such an approach would be particularly useful to devise high-accuracy specialised CR tools.

### Comparison with state of the art

For completeness purposes, we present in Table 2 a comparative overview of the results achieved by our approach against the state of the art systems, using results previously published by [9] for Neji and by [13] in their very recent study on off-the-shelf CR systems, for NCBO Annotator, MetaMap and ConceptMapper. Overall, the best performing system is ConceptMapper—as reported also by [13]. With a single exception in the case of GO_CC, where our approach has outperformed all other systems, including ConceptMapper (although with only 0.01 F1), ConceptMapper has consistently achieved the best performance on CL, ChEBI, PRO and SO. Results on GO_BMPF are comparable only between Neji and our approach, because [13] split GO_BPMF in two individual categories: biological processes (BP) and molecular functions (MF). Initially Neji has outperformed our baseline in GO_BPMF because of their use of specialised dictionaries to support the CR process. However, the information gain has increased the F-Score to 0.40, i.e., with 0.05 more than the result achieved by Neji. A similar behaviour is present also in PRO, where case sensitivity has led to an increase in F-Score that outperformed Neji with 0.05 F1.

**Table 2:** Comparison with state of the art systems: Neji, NCBO Annotator, MetaMap, ConceptMapper.

Ontology	Neji			NCBO Annotator			MetaMap			ConceptMapper			Our best result		
	P	R	F1	P	R	F1	P	R	F1	P	R	F1	P	R	F1
CL	0.63	0.65	0.64	0.76	0.20	0.32	0.61	0.80	0.69	0.88	0.78	**0.83**	0.85	0.72	*0.78*
GO_CC	0.75	0.66	0.70	0.75	0.27	0.40	0.67	0.73	0.70	0.92	0.66	0.77	0.84	0.73	**0.78**
GO_BPMF	0.44	0.29	0.35	-	-	-	-	-	-	-	-	-	0.48	0.34	**0.40**
ChEBI	0.49	0.36	0.33	0.70	0.46	**0.56**	0.36	0.50	0.42	0.55	0.56	**0.56**	0.48	0.55	*0.51*
PRO	0.46	0.39	0.42	0.49	0.51	0.50	0.39	0.34	0.36	0.57	0.57	**0.57**	0.42	0.53	0.47
SO	-	-	-	0.63	0.33	0.44	0.47	0.54	0.50	0.56	0.57	**0.56**	0.51	0.52	*0.52*

Bolded values denote the best F-Score across all methods. ConceptMapper outperforms all approaches on all ontologies. Our method achieves an increased efficiency against the other three systems on three ontologies: CL, GO_CC and SO, in addition to outperforming Neji on GO_BPMF

If we are to consider the comparison between “pure” biomedical CR systems (i.e., excluding ConceptMapper), our approach outperforms all the other systems in most cases. In CL, our best result (0.78 F1) is better with 0.09 F1 than MetaMap and 0.14 F1 than Neji. Similarly, in GO_CC we observe an improved F1 with 0.08 when compared to MetaMap and Neji. In the context of these two ontologies, the NCBO Annotator achieves particularly low results. The gap in F1 decreases on more complex ontologies, such as ChEBI, PRO and SO. Our approach is better than MetaMap on ChEBI with 0.08 F1 and on PRO with 0.11 F1—in these two cases, the NCBO Annotator either performs on par with ConceptMapper, e.g., on ChEBI, or outperforms our approach—e.g., on PRO—with 0.03 F1. Finally, on SO, our approach achieves, again, a better performance with 0.02 F1 and 0.08 F1, respectively, when compared to MetaMap and the NCBO Annotator (here, a comparison with Neji was not possible as [9] have not tested it on SO).

It is almost impossible to draw a clear conclusion on the reasons behind the variation in performance between the different systems on the above ontologies. There are, however, a few aspects that are worth mentioning:

- Both MetaMap and ConceptMapper are highly configurable systems, which can be tailored for particular tasks and ontology concept shapes. Two important parameters featured by these systems and that played an important role in improving CR performance are: *allow concept gaps* and *allow nested concepts* (or *find all matches*). As per its name, the former enables systems to find textual mentions that contain gaps and associate them with concepts—e.g., “*cells in … epithelial … regions*” referring to CL:0000066 (*epithelial cell*). The latter acts as a filter and restrains the list of candidate mentions only to the longest matches—e.g., mapping only CL:0000066 (*epithelial cell*) to “*epithelial cells*” and ignoring the mapping of CL:0000000 (*cell*) to “*cells*” in the context of “*epithelial cells*”. Neither the NCBO Annotator, nor our approach are able to deal with concept gaps, and both of them aim to find all concepts, which may lead to false positives—in particular in CL.
- Another parameter, configurable only in ConceptMapper, is the set of synonyms to be taken into account when performing CR—i.e., using no / EXACT / RELATED / ALL synonyms. This has a direct impact on the resulting performance as it may lead, similar to the “*find all matches*”, to false positives. Except for ConceptMapper, all other systems have a less fine-grained manner of dealing with synonyms, allowing their simple bulk inclusion or exclusion (in our case, we have just included all). [13] have tested multiple configurations for ConceptMapper and listed the results achieved by the best setting—which has been different for different ontologies—e.g., EXACT only for SO and ChEBI or ALL for PRO.

## Conclusion

In this article we have presented a concept recognition approach that exploits two facets rarely used in this process: case sensitivity and token information gain. Case sensitivity is assessed by considering the distribution of so-called case-sensitive tokens in the underlying ontology. Information gain, on the other hand, is computed using the divergence from randomness and mutual information of the tokens present in the lexical representations (e.g., labels, synonyms) of the ontological concepts. Experimental results have shown that, subject to the underlying token distribution, these two components lead to improvements in the CR efficiency in ontology-specific contexts. Consequently, they can be adopted as complementary strategies for generating alternative labels sets.

Future work will focus on integrating distributional semantics in the computation of the information gain and mutual information and on providing an option to learn its underlying parameters via a supervised or semi-supervised method. We will also more systematically explore the complementarity of our proposals with other proposals exploiting term evidence and specificity in the context of ontological structures.

## Supporting Information

S1 Fig Distribution of pairwise Mutual Information in the Cell Ontology. (TIFF)

S2 Fig Distribution of pairwise Mutual Information in GO Cellular Component. (TIFF)

S3 Fig Distribution of pairwise Mutual Information in ChEBI. (TIFF)

S4 Fig Distribution of pairwise Mutual Information in GO Biological Process & Molecular Function. (TIFF)

S5 Fig Distribution of pairwise Mutual Information in the Protein Ontology. (TIFF)

S6 Fig Distribution of pairwise Mutual Information in the Sequence Ontology. (TIFF)

S7 Fig Variation of the F-Score with the independence interval in the Cell Ontology. (TIFF)

S8 Fig Variation of the F-Score with the independence interval in GO Cellular Component. (TIFF)

S9 Fig Variation of the F-Score with the independence interval in GO Biological Process & Molecular Function. (TIFF)

S10 Fig Variation of the F-Score with the independence interval in ChEBI. (TIFF)

S11 Fig Variation of the F-Score with the independence interval in the Protein Ontology. (TIFF)

S12 Fig Variation of the F-Score with the independence interval in the Sequence Ontology. (TIFF)
