# Supplementary figures and images for: Assessing the Impact of Case Sensitivity and Term Information Gain on Biomedical Concept Recognition
Source: PLoS One. 2015 Mar 19;10(3):e0119091. doi: 10.1371/journal.pone.0119091 (PMC4366016; doi:10.1371/journal.pone.0119091)

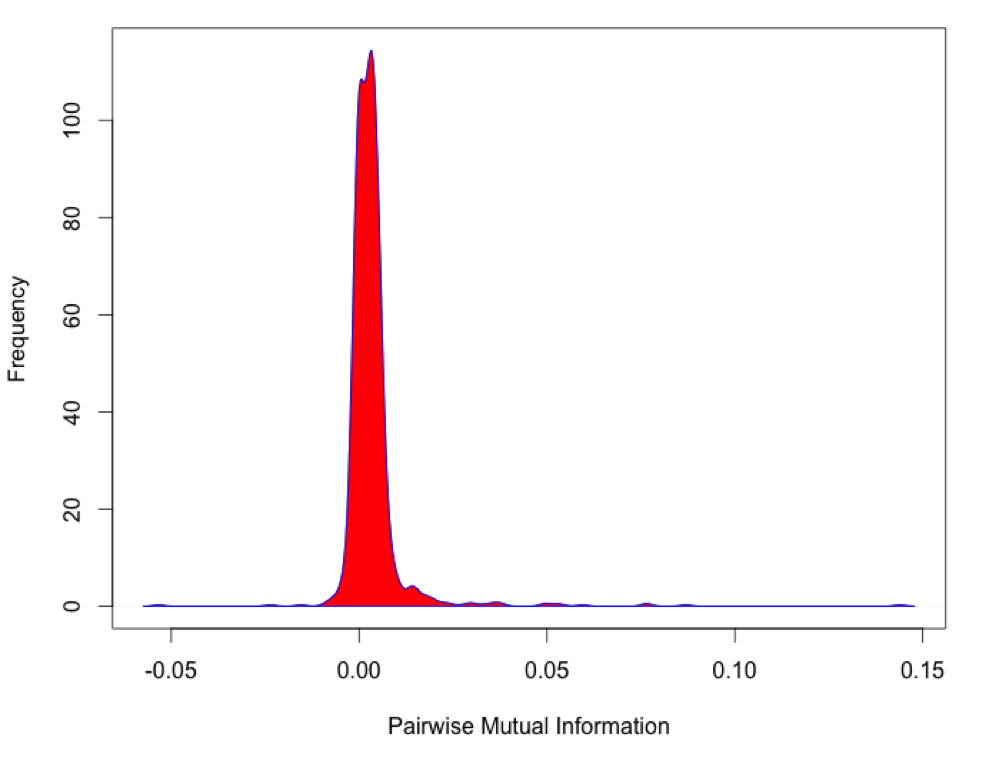

Supplement: S1 Fig — (TIFF) [file pone.0119091.s001.tiff]

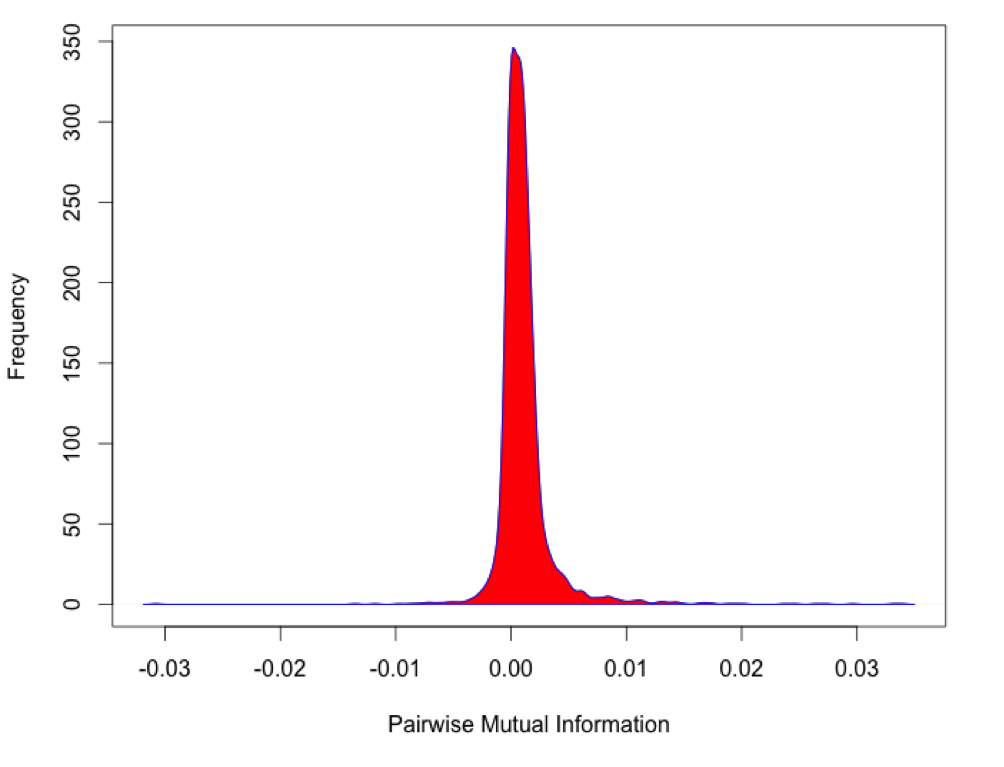

Supplement: S2 Fig — (TIFF) [file pone.0119091.s002.tiff]

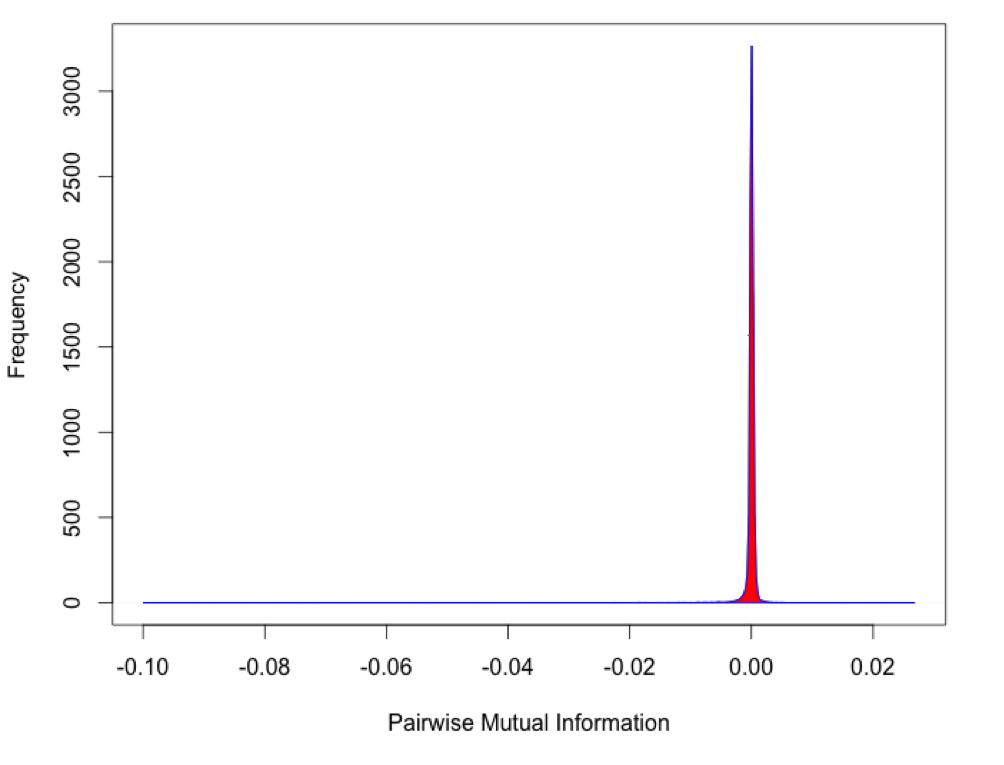

Supplement: S3 Fig — (TIFF) [file pone.0119091.s003.tiff]

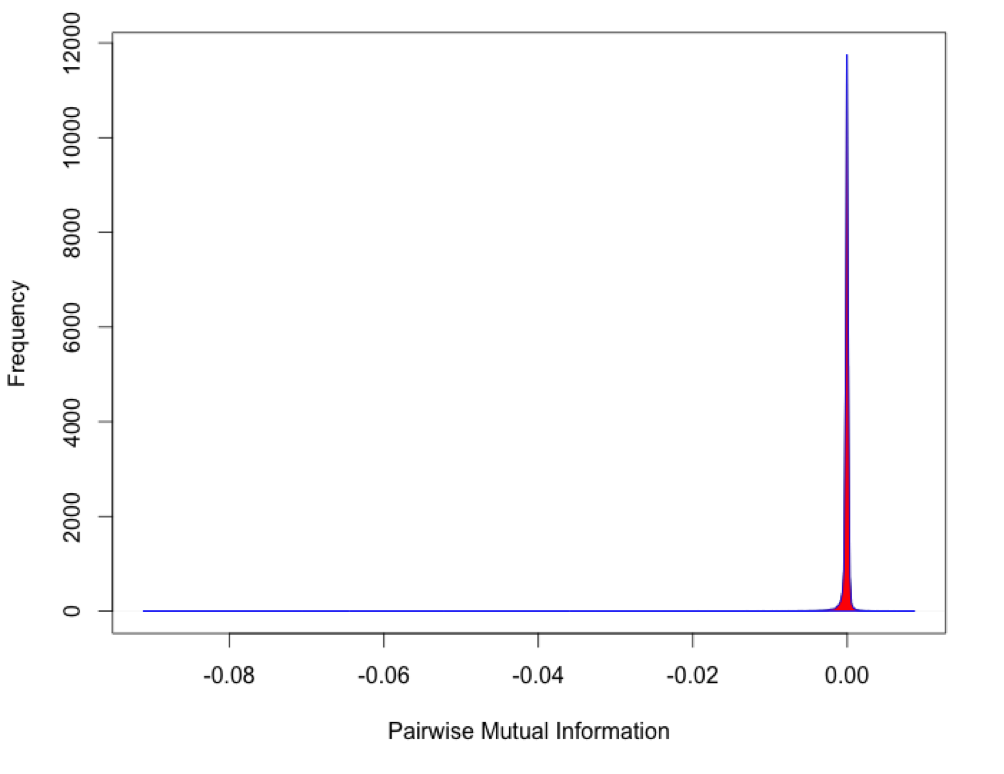

Supplement: S4 Fig — (TIFF) [file pone.0119091.s004.tiff]

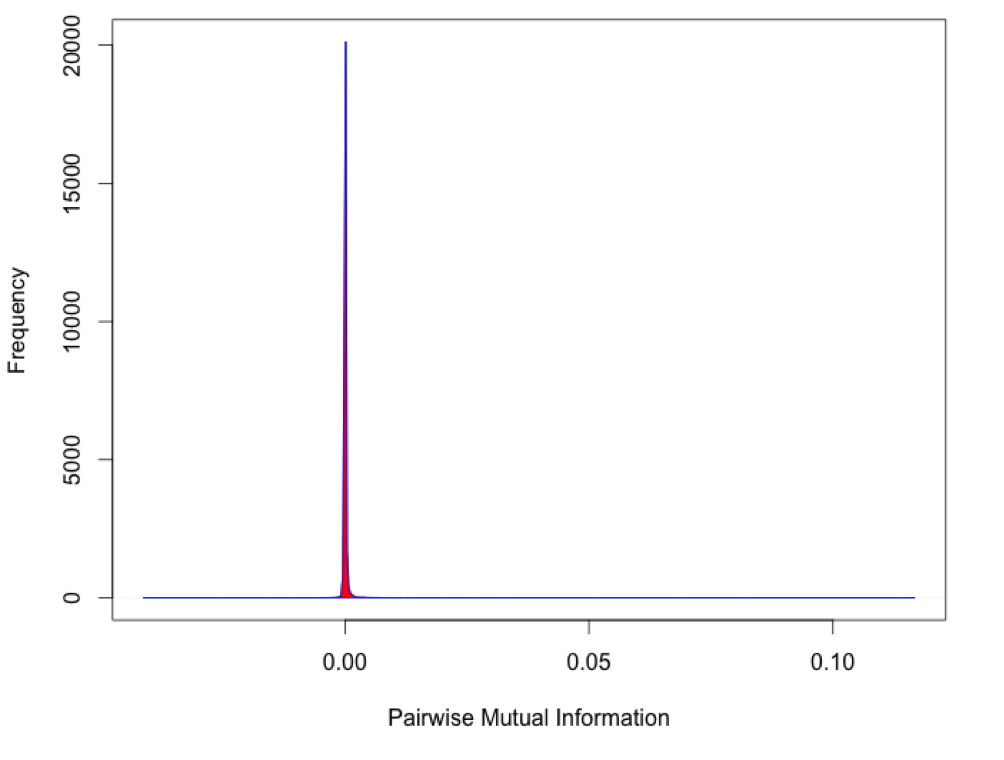

Supplement: S5 Fig — (TIFF) [file pone.0119091.s005.tiff]

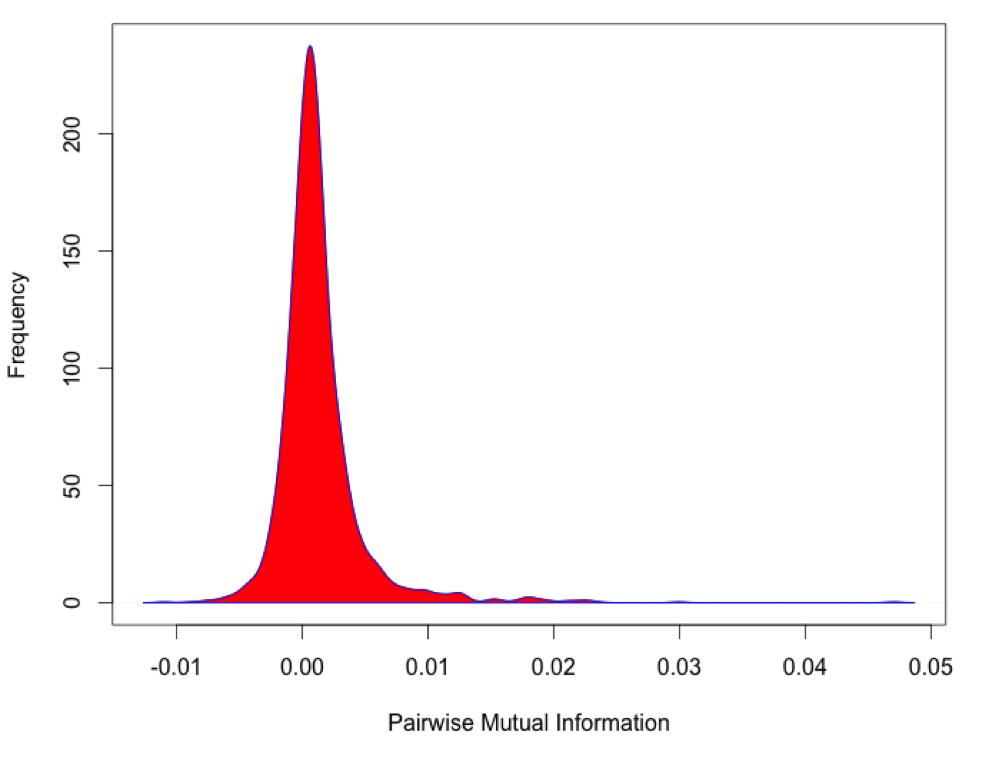

Supplement: S6 Fig — (TIFF) [file pone.0119091.s006.tiff]

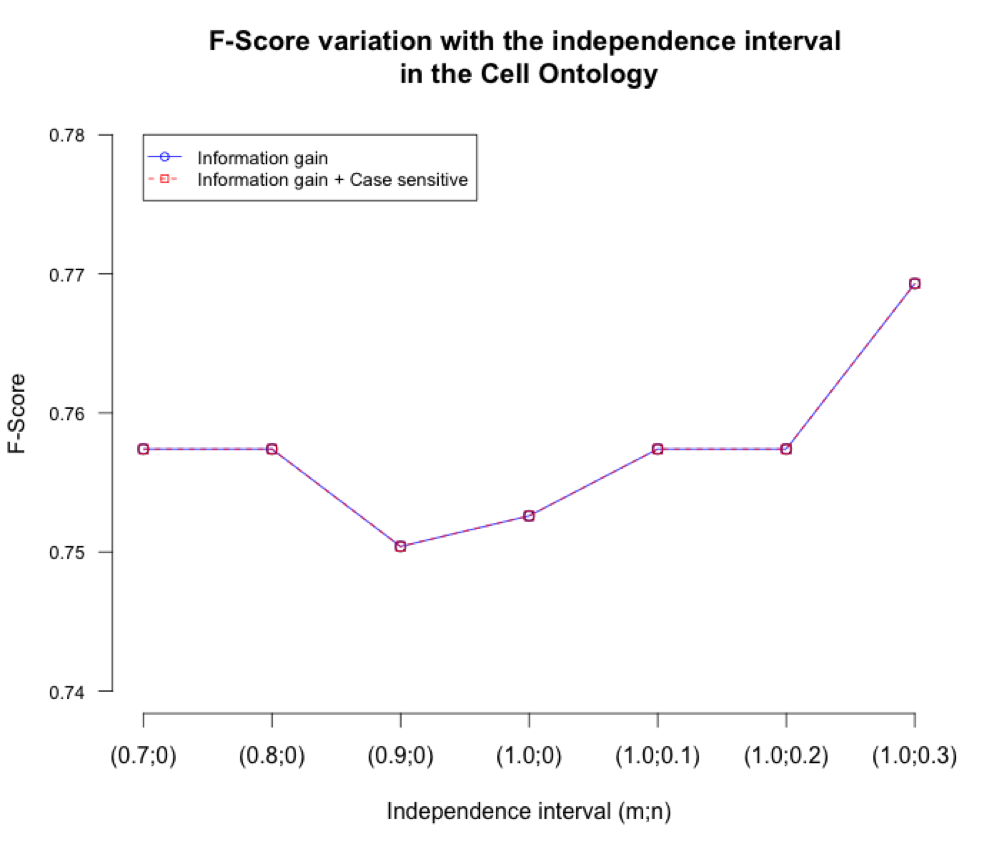

Supplement: S7 Fig — (TIFF) [file pone.0119091.s007.tiff]

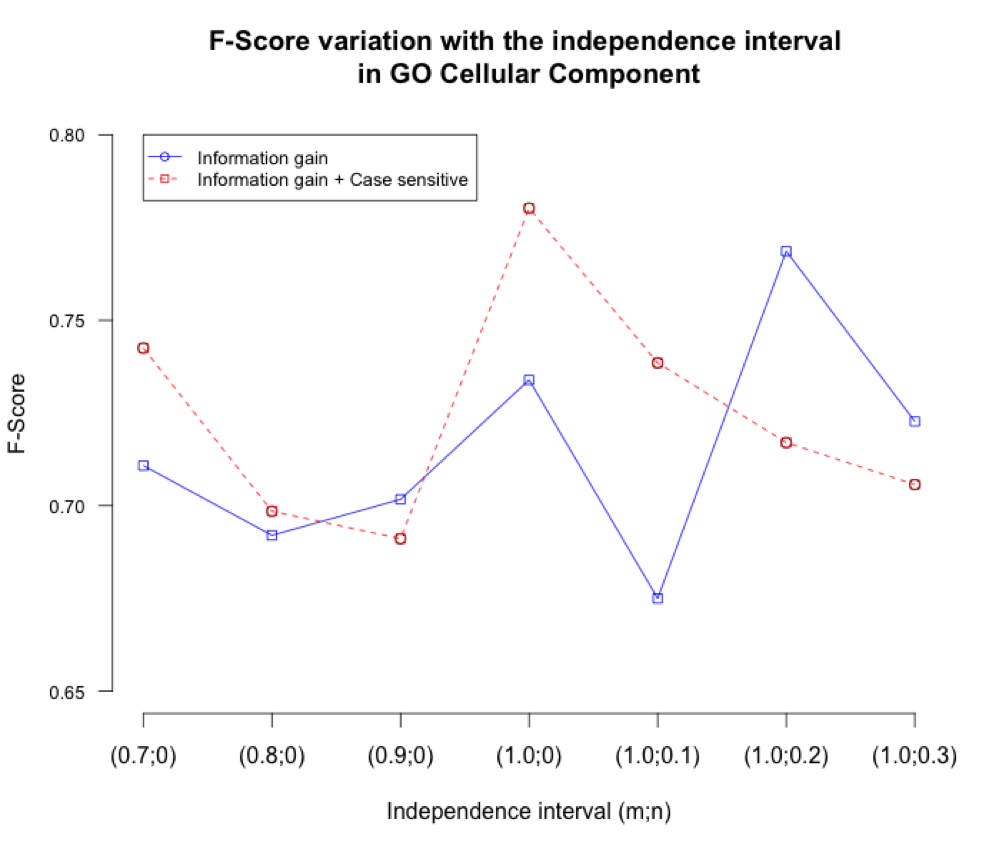

Supplement: S8 Fig — (TIFF) [file pone.0119091.s008.tiff]

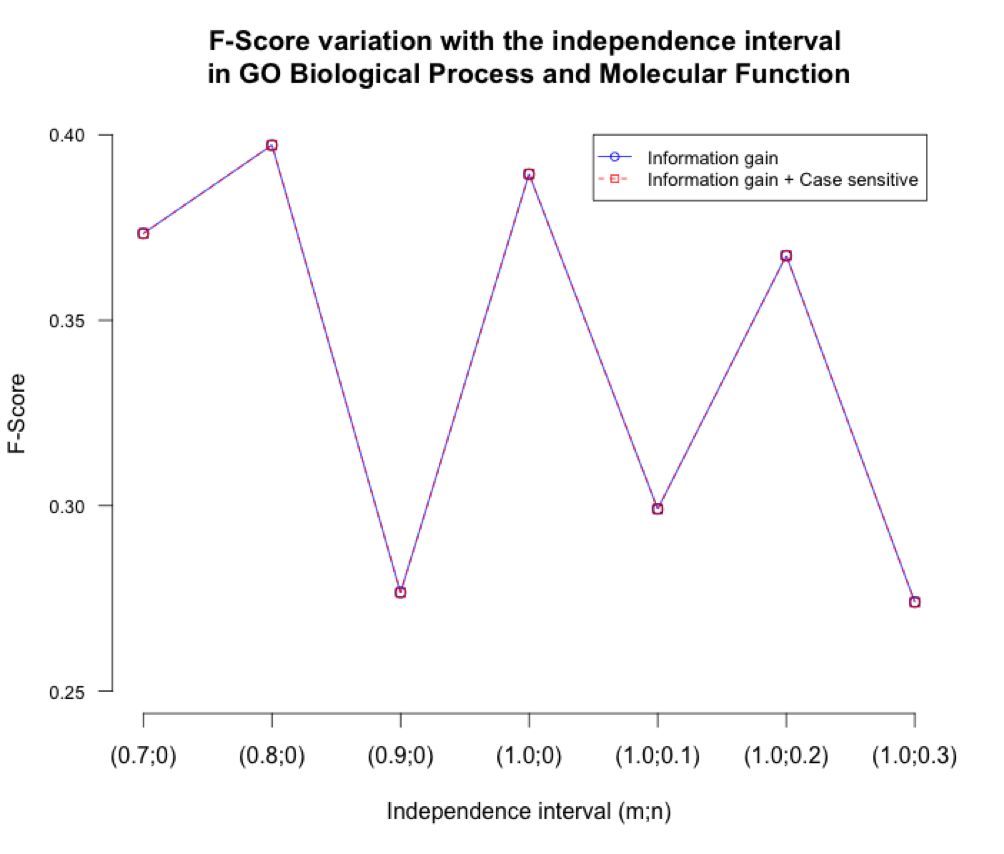

Supplement: S9 Fig — (TIFF) [file pone.0119091.s009.tiff]

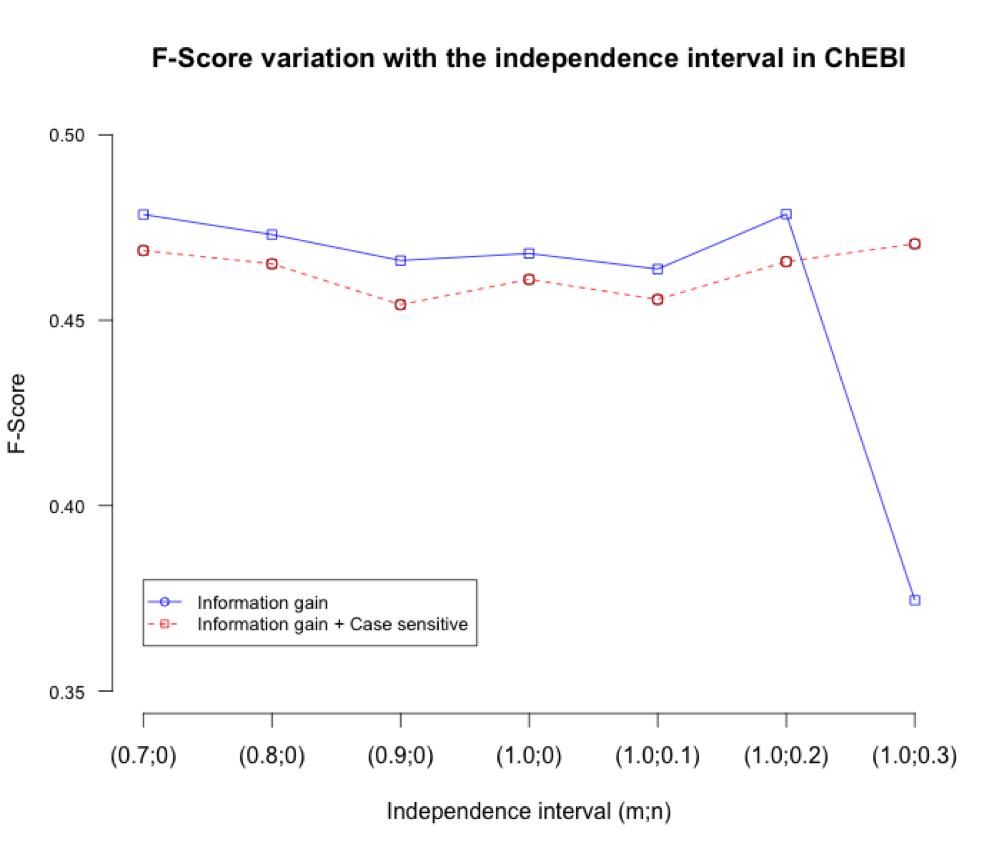

Supplement: S10 Fig — (TIFF) [file pone.0119091.s010.tiff]

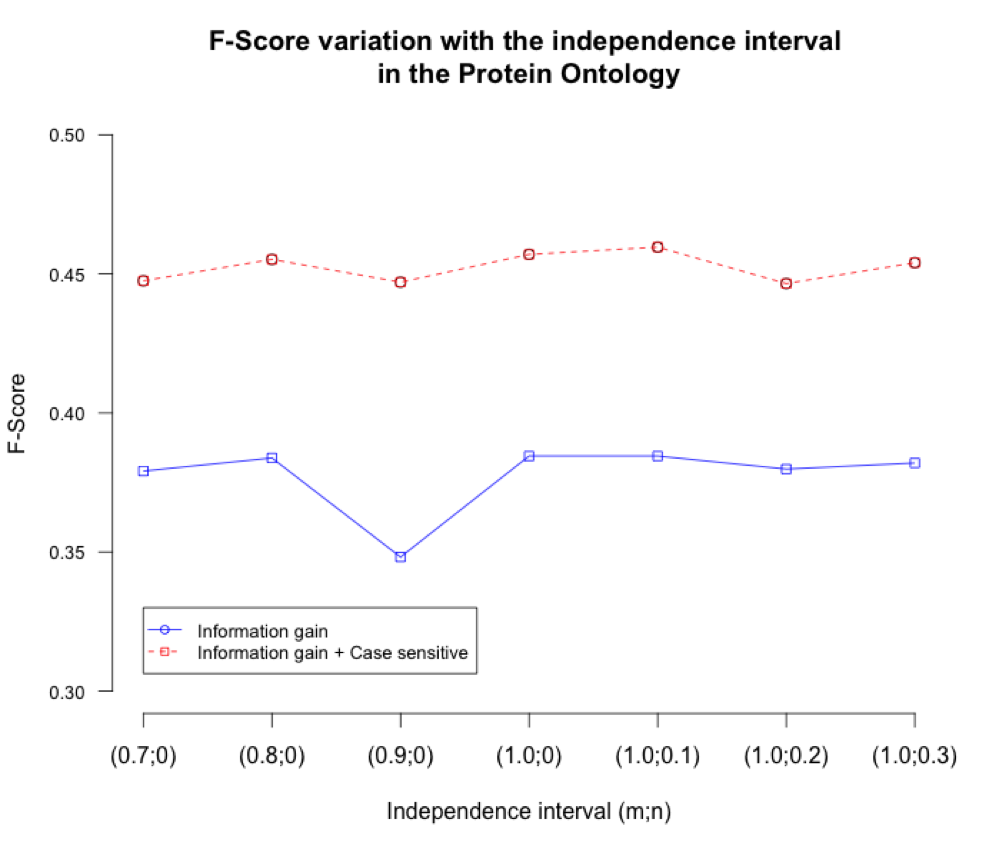

Supplement: S11 Fig — (TIFF) [file pone.0119091.s011.tiff]

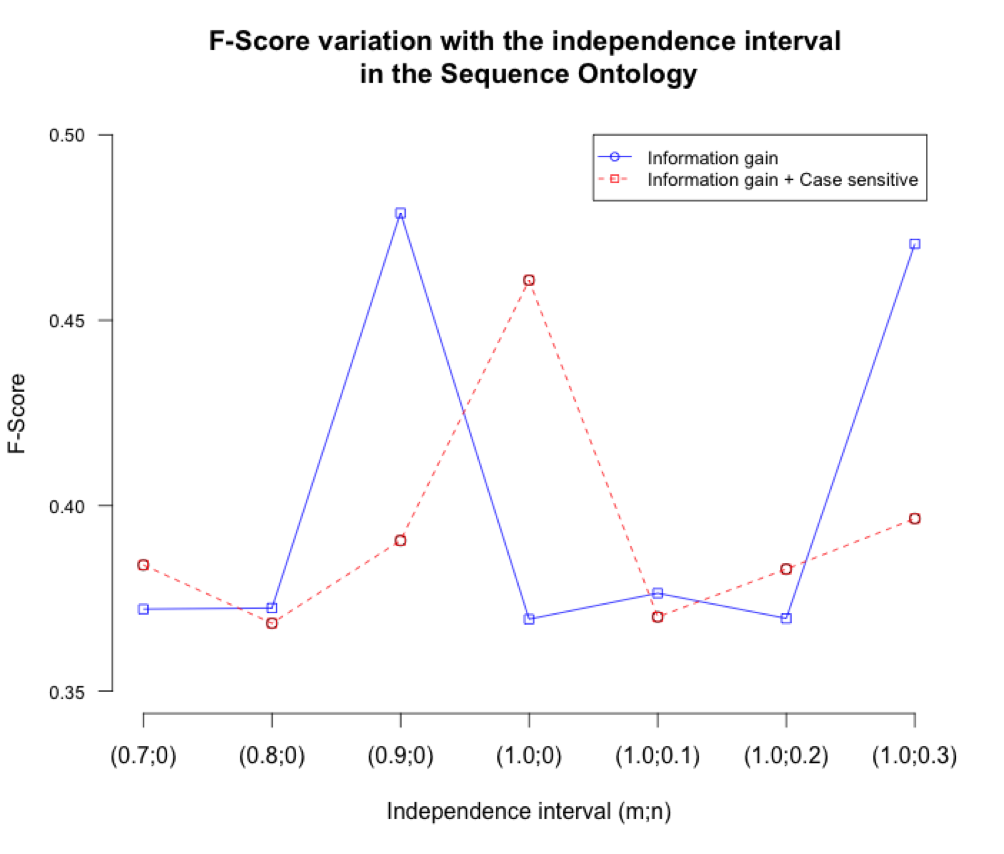

Supplement: S12 Fig — (TIFF) [file pone.0119091.s012.tiff]
